# Influence of opacifiers on dimensional stability and detail reproduction of maxillofacial silicone elastomer

**DOI:** 10.1186/1475-925X-9-85

**Published:** 2010-12-16

**Authors:** Marcelo C Goiato, Marcela F Haddad, Mário A C Sinhoreti, Daniela M dos Santos, Aldiéris A Pesqueira, Amália Moreno

**Affiliations:** 1 Department of Dental Materials and Prosthodontics, Araçatuba Dental School, UNESP. Rua José Bonifácio, 1193. CEP 16015-050. Araçatuba, SP, Brazil; 2 Department of Prosthodontics and Periodontology, Piracicaba Dental School, University of Campinas, UNICAMP. CEP 13414-903. Piracicaba, SP, Brazil

## Abstract

**Background:**

We evaluated the influence of chemical disinfection and accelerated aging on the dimensional stability and detail reproduction of a silicone elastomer containing one of two opacifiers.

**Methods:**

A total of 90 samples were fabricated from Silastic MDX 4-4210 silicone and divided into groups (n = 10) according to opacifier content (barium sulfate or titanium dioxide) and disinfectant solution (neutral soap, Efferdent, or 4% chlorhexidine). The specimens were disinfected 3 times per week during 60 days and then subjected to accelerated aging for 1008 hours. Dimensional stability and detail reproduction tests were performed after specimens' fabrication (baseline), chemical disinfection and periodically during accelerated aging (252, 504, and 1008 hours). The results were analyzed using 3-way repeated-measures ANOVA and the Tukey HSD test (α = 0.05).

**Results:**

All groups exhibited dimensional changes over time. The opacifier (p = .314), period (p < .0001) and their interactions (p = .0041) affected the dimensional stability of the silicone. Statistical significant dimensional differences occurred between groups with (0.071) and without opacifiers (0.053). Accelerated aging influenced the dimensional stability of the samples. All groups scored 2 in the detail reproduction tests, which represents the fully reproducing of three test grooves with accurate angles.

**Conclusions:**

Incorporation of opacifiers alters the dimensional stability of silicones used in facial prosthetics, but seems to have no influence on detail reproduction. Accelerated aging is responsible for most of the dimensional changes in Silastic MDX4 4210, but all dimensional changes measured in this study remained within the limits of stability necessary for this application.

## Background

In contemporary society where beauty is considered essential, patients with facial mutilations due to congenital malformations, oncologic surgery, or trauma are often marginalized [1-3]. In view of this reality, the goal of facial prosthetic technology is to offer individuals' aesthetic and comfort while improving their self-esteem and quality of life [4,5].

To be considered aesthetically pleasing, the facial prosthesis should accurately reproduce the skin color and anatomic detail of the patient and to closely approximate the margins of the defect. Therefore, it is necessary to use a material that can faithfully reproduce the contours of the defect and at the same time resist to deterioration in texture and be dimensionally stable even after disinfection and aging.

The dimensional stability is an important property of a material to provide prosthesis's fitting over time, protection of the bloody tissues as well as aesthetic [6,7]. The detail reproduction is directly related to the prosthesis's aesthetic since the material should faithfully reproduce the details of the patient's face such as wrinkles and sulcus in order to provide a more natural appearance to the prosthesis [6,7].

Elastomeric silicones were introduced in maxillofacial prosthetics field during the 1960s and became the material of choice [6-11]. Limitations of using silicone to fabricate facial prostheses include color instability and rapid degradation of the material [12].

Recently, the literature has shown that the incorporation of opacifier in the silicone matrix increases material durability [13-16], and keeps the prosthesis esthetically pleasing in relation to color stability for a long time. A great deal of research has been devoted to developing processes to incorporate opacifiers into polymers, providing a new class of materials that offers at the same time strength and flexibility of an organic polymer matrix [13-16]. Opacifiers are rigid and have a higher shear modulus when compared to pure silicone elastomer [17]. The enhanced properties obtained by adding opacifiers to a polymer may be attributed to the higher surface energy and chemical reactivity of the particles, allowing them to interact with the elastomer matrix to form a 3-dimensional network within the silicone structure [18-20]. They improve the physical and optical properties of the organic polymer, as well as provide resistance to stress-induced cracking and aging [21]. However, the influence of opacifiers on physical properties such as dimensional stability and fidelity of detail reproduction of silicones has not been fully investigated.

Several methods to evaluate dimensional stability have been described in the literature. Traditional measurement methods such as compasses, calipers, and linear microscopes are common, but recently computational methods can offer high precision. Such methods have been widely used to simulate masticatory efforts and can also be adapted to perform stability measurements. The AutoCAD software was developed to create graphics in engineering and physics [22] and is currently applied in medicine and dentistry both in clinical [23] and laboratory [24] procedures. This software can provide quick and accurate measurements of digitally scanned samples.

The aim of this study was to investigate the dimensional stability and detail reproduction of facial silicone prostheses fabricated with and without the incorporation of opacifiers, and to determine the influence of disinfection and accelerated aging on these parameters.

## Methods

The samples were fabricated using Silastic MDX4-4210 (Dow Corning Corporation, Midland, MI, USA). Barium sulfate (Wako, Osaka, Japan) and titanium dioxide (Homeofar, Catanduva, SP, Brazil) were used as opacifiers.

A cylindrical metal mold with 30 mm in diameter and 3 mm in thickness was used to cats the samples [6,12]. A total of 90 samples were divided into 9 groups (n = 10) according to opacifier content (none (L), barium sulfate (Ba), or titanium dioxide (Ti)) and disinfection method (soap (S), Efferdent (E), or chlorhexidine (C)).

The materials were weighed using a precision digital scale (BEL Equipamentos Analítico, Piracicaba, SP, Brazil). The opacifiers were added at a concentration of 0.2wt% of the silicone.

Silicone was formulated and mixed according to the manufacturer's instructions at 23 ± 2°C. The opacifiers were added to the silicone using a stainless steel spatula on a glass plate to obtain a homogenous mass. After manipulation, the mixture was placed in the mold and cured at room temperature during 3 days according to the manufacturer's instructions [6,9,25]. Sample was carefully removed from the mold to avoid distortions [6,11]. Dimensional stability and detail reproduction tests were carried out initially, after 60 days of disinfection, and after different times of accelerated aging (252, 504, and 1008 hours).

**Figure 1:**
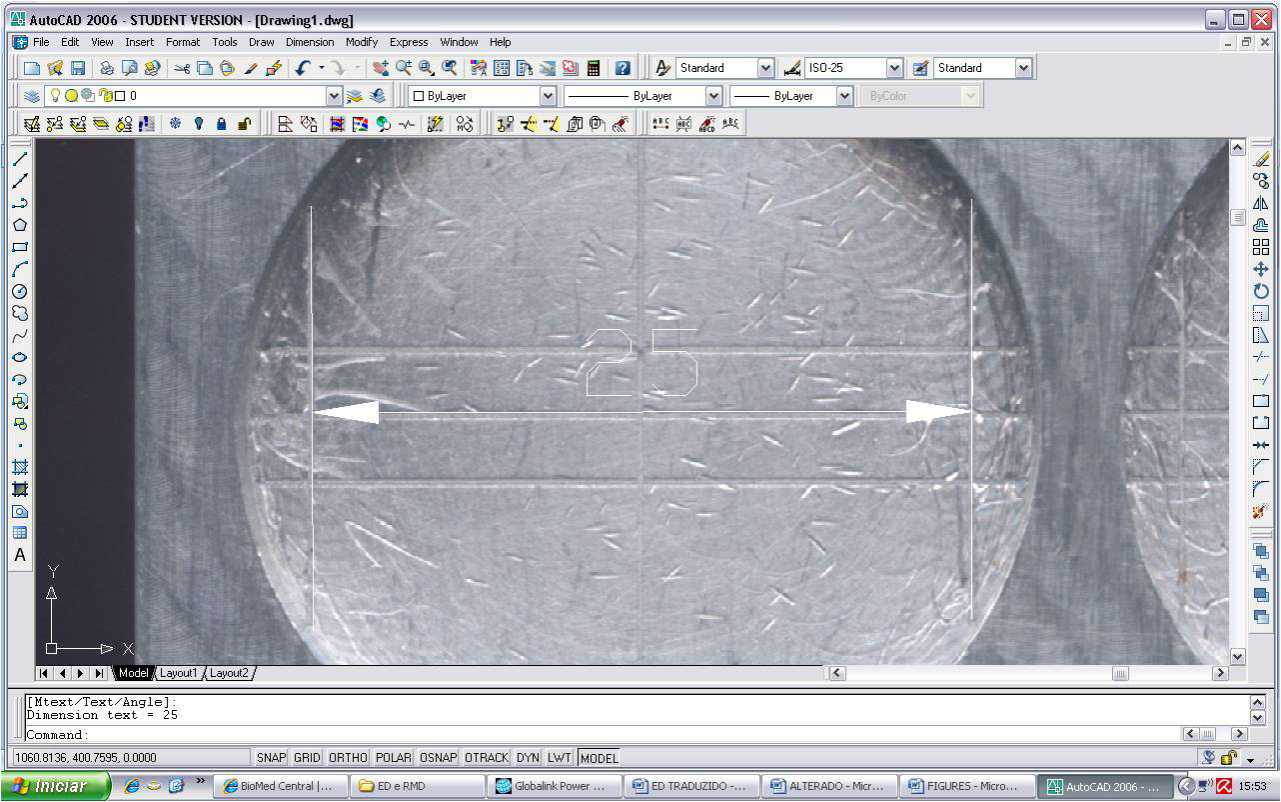
**Matrix measurement using AutoCAD software**. Measurement of the original distance between edges C and D of the matrix.

**Figure 2:**
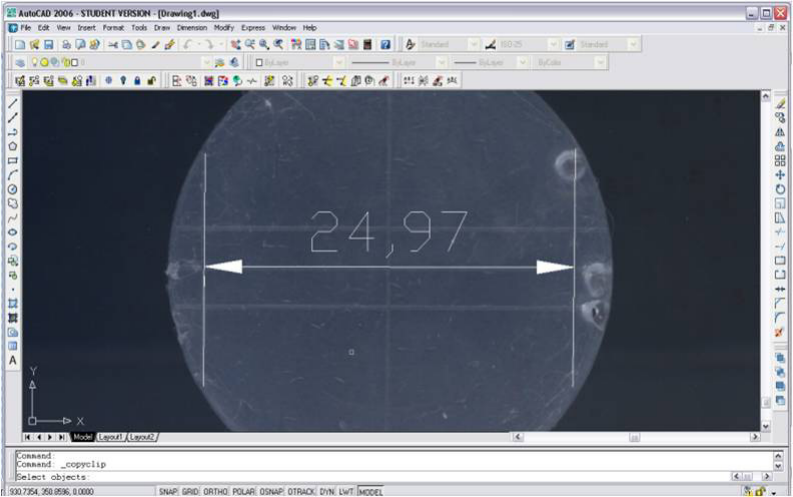
**Sample measurement using AutoCAD software**. Measurement of the distance between edges C' and D' of the samples.

For the dimensional stability test, samples were digitalized using a scanner (Genius, Langenfeld, Nordrhein-Westfalen, Germany) with a resolution of 800 dpi. In order to standardize the measurement, a metallic block with two vertical lines (C-D) 25 mm apart was scanned with each sample. The measurements were performed using AutoCAD 2008 software (R16 version N63.0, Autodesk Inc, San Rafael, CA, USA). Both sample's and matrix's images were imported into the software using the "Raster image" tool [6,24]. This tool allows working with bitmap images within vector-based files constructed using straight lines. The vertical lines of the block were used as a 25-mm dimensional reference (C - D) within the software (Figure 1). Vertical lines inscribed on the samples (C' - D') were compared against this reference (Figure 2).

Each sample was measured three times. The following formula was applied to determine dimensional changes [6,26]:

$$\text{D}\text{i}\text{m}\text{e}\text{n}\text{s}\text{i}\text{o}\text{n}\text{a}\text{l}\;\text{c}\text{h}\text{a}\text{n}\text{g}\text{e}\;\text{\%}=\frac{\left(\text{B}-\text{A}\right)}{\text{A}}\times 100$$

*A = original distance of the block between edges C and D = 25 mm*.

B = distance between edges C' and D' in the samples

In the detail reproduction test, the angular accuracy of three grooves (20 μm, 50 μm and 75 μm wide) molded in each sample was recorded. Detail reproduction was examined using a stereo microscope (Olympus, Tokyo, Japan) under low-angle illumination at 13× magnification. To classify the accuracy of detail reproduction, the scores suggested by Goiato et al. [6] were used as described below:

X: no groove reproduction;

0: full reproduction of two of the three grooves;

1: full reproduction of the 3 grooves, with inaccurate angles

2: full reproduction of the 3 grooves, with accurate angles.

Samples were disinfected 3 times per week during 60 days [6,8-10,12]. Ten samples of each group were disinfected with Efferdent [6-9,12], ten with neutral soap [6,10,12], and ten with 4% chlorhexidine gluconate (Naturativa, Araçatuba, SP, Brazil) [18].

The groups disinfected with water and neutral pH soap (Johnson & Johnson, São José dos Campos, SP, Brazil) were rubbed with the fingertips during 30 seconds, then rinsed with water [6,8,9,12]. The samples disinfected with Efferdent (Pfizer Consumer Health, Morris Plains, NJ, USA) were immersed in a solution containing Efferdent tablets dissolved in 250 mL of warm water during 15 min [6,8,9]. Samples disinfected with chlorhexidine were immersed in this solution for 10 minutes and then rinsed with water [12].

Accelerated aging treatments were carried out using an aging chamber (Equilam, Diadema, SP, Brazil) [13,25,27]. Each aging cycle lasted twelve hours. In the first eight hours, the samples were exposed to ultraviolet light at a temperature of 60 ± 3°C. In the remaining four hours, samples were subjected to oxygen-saturated distilled water condensate in the absence of light at a temperature of 45 ± 3°C. The samples underwent 1008 hours of accelerated aging.

Both disinfection and accelerated aging processes simulated 1 year of patient's clinical use of the silicone [25,27].

The effect of opacifiers, disinfection, and aging on dimensional stability (interactions among these factors) was analyzed by 3-way repeated-measures analysis of variance (ANOVA). Means were compared using the Tukey HSD test (α = 0.05). As all of the samples achieved the same detail reproduction score, no statistical analysis was performed for this variable.

## Results

Table 1 lists the means and standard deviations of the dimensional change values of the silicone for each period of analysis. All groups exhibited dimensional changes over the course of the experiment (table 1). Both the opacifiers (*P *= 0.314) and time (*P *< 0.0001) statistically affected the dimensional stability of the silicone (table 2). The interaction between period and opacifier type (*P *= .0041) was statistically significant (tables 2 and 3).

**Table 1:** Mean values (SDs) of dimensional change in absolute value (%).

	PERIODS				
**GROUP**	**Initial**	**60 days**	**Aging (252 hrs)**	**Aging (504 hrs)**	**Aging (1008 hrs)**

LS	0.010 (0.003)	0.048 (0.010)	0.048 (0.011)	0.064 (0.015)	0.064 (0.010)
LE	0.012 (0.006)	0.036 (0.013)	0.056 (0.019)	0.060 (0.013)	0.060 (0.012)
LC	0.010 (0.002)	0.060 (0.009)	0.064 (0.011)	0.104 (0.008)	0.104 (0.019)
BaS	0.020 (0.005)	0.048 (0.006)	0.096 (0.012)	0.096 (0.018)	0.116 (0.019)
BaE	0.016 (0.004)	0.064 (0.007)	0.080 (0.009)	0.080 (0.007)	0.088 (0.007)
BaC	0.020 (0.007)	0.036 (0.007)	0.052 (0.010)	0.116 (0.023)	0.144 (0.027)
TiS	0.024 (0.005)	0.044 (0.007)	0.068 (0.009)	0.068 (0.012)	0.108 (0.014)
TiE	0.016 (0.004)	0.060 (0.006)	0.072 (0.012)	0.092 (0.012)	0.092 (0.018)
TiC	0.008 (0.004)	0.028 (0.007)	0.052 (0.012)	0.128 (0.020)	0.200 (0.024)

**Table 2:** Results of 3-way repeated-measures ANOVA for dimensional changes.

Source	*df*	SS	MS	F	*P*
Disinfectant	2	0.020	0.010	2.144	.1238
Opacifier	2	0.034	0.017	3.613	.0314*
Disinfectant × opacifier	4	0.001	0.000	0.067	.9915
Between subjects	81	0.382	0.005		
Periods	4	0.365	0.091	47.692	< .0001*
Periods × disinfectant	8	0.036	0.004	2.349	.0826
Periods × opacifier	8	0.044	0.006	2.886	.0041*
Periods × disinfectant × opacifier	16	0.016	0.001	0.530	.9305
Within subjects	324	0.620	0.002		

*P *< .05 difference statistically significant.

**Table 3:** Mean values of dimensional change in absolute value (%) at several elapsed times, and opacifier contents, independent of disinfectant.

	PERIODS				
**OPACIFIER**	**Initial**	**60 days**	**Aging (252 hrs)**	**Aging (504 hrs)**	**Aging (1008 hrs)**

Colorless	0.011 Aa	0.048 Aab	0.056 Aab	0.076 Ab	0.076 Ab
Barium	0.019 Aa	0.049 Aab	0.076 Ab	0.097 Abc	0.116 Bbc
Titanium	0.016 Aab	0.044 Ab	0.064 Abc	0.096 Ac	0.133 Bcd

Different uppercase letters in column denote statistically significant difference (*P *< .05).

Different lowercase letters in lines denote statistically significant difference (*P *< .05).

**Figure 3:**
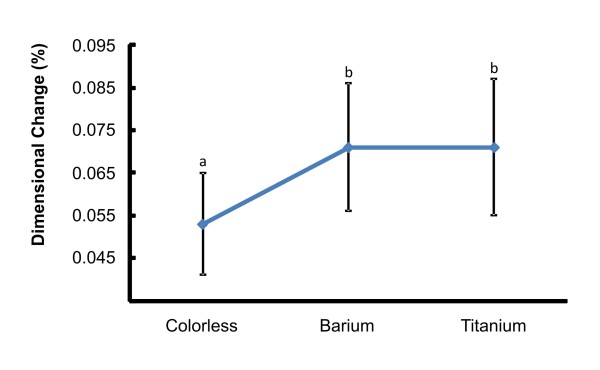
**Mean values of dimensional change in absolute value (%) for each opacifier, regardless of the elapsed period or disinfection method**. * Different letters denote statistically significant differences between opacifiers (*P *< 0.05).

**Figure 4:**
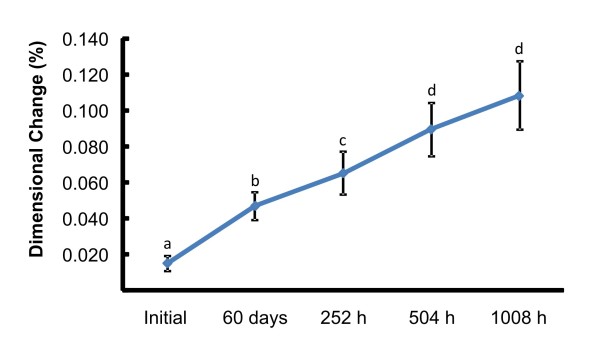
**Mean values of dimensional change in absolute value (%) for each period of time, independent of disinfection or opacifier**. * Different letters denote statistically significant differences between times (*P *< 0.05).

Groups containing opacifiers (BaS, BaE, BaC = 0.071; TiS, TiE, TiC = 0.071) exhibited significantly higher dimensional changes (LS, LE, LC = 0.053) (Figure 3). Statistically significant dimensional changes occurred among the initial measurement, after 60 days of disinfection and after 1008 hours of accelerated aging (Figure 4).

All groups scored 2 in the detail reproduction test, showing fully reproduction of the three test grooves with accurate angles.

## Discussion

All groups exhibited dimensional changes over the experiment (table 1). At the initial period, the dimensional changes may be attributed to the polymerization shrinkage of the elastomer [6,8,9,11,12]. The increased dimensional changes observed during other periods (60 days, 252, 504, and 1008 hours) can be explained by the release of formaldehyde, a byproduct of the silicone's curing process [6,9,28]. It is well known that elastomers can contract during polymerization. Anusavice [28] stated that there are five main reasons to promote dimensional changes in elastomeric materials: polymerization shrinkage, byproduct release during condensation reactions, thermal shrinkage due to temperature changes, sorption after exposure to water, disinfectants, or high humidity environments for long periods, and incomplete elastic deformation recovery due to viscoelastic behavior.

The disinfectant type did not significantly affect the dimensional stability of the silicone (table 2). Similar results were reported by Guiotti et al. [12], who used a scanning electron microscope to evaluate the marginal deterioration of facial prostheses after chlorhexidine disinfection. The authors did not observe any significant alteration. Goiato et al. [6] examined the dimensional alteration and detail reproduction of two silicones used in facial prostheses after disinfection with neutral soap and alkaline peroxide effervescent tablets. They did not find any significant alteration of these properties. Similar results were obtained in another study [29], in which the immersion of silicone impression material in various disinfectants did not alter the details of the impression.

The incorporation of opacifiers in the silicone significantly increased the dimensional changes of the samples (table 3 and Figure 3) regardless of elapsed time or disinfectant. This can be attributed to the fact that opacifiers present high surface energy and chemical reactivity, causing their nanosized particles to agglomerate [30]. When the elastomer is subjected to external forces, the agglomerated particles act as stress-concentrating centers in the silicone matrix, thereby decreasing the mechanical strength of the material [31] and potentially affecting the dimensional stability over time.

It appears that period of time is responsible for the highest dimensional changes in all groups (table 3 and Figure 4). This can be explained by the continuous extended polymerization of elastomeric materials [8,28] with the release of formaldehyde [8]. Most polymers contain aromatic rings and C = C bonds in their structures, which can absorb ultraviolet light during accelerated aging [28]. When the polymer molecule absorbs ultraviolet light, this energy creates instability in the molecular structure. The excess of energy can be dissipated through several pathways, for instance transfer of the excitation to another molecule [28]. These functional groups may return to their ground state in steps, re-emitting the excess of energy at longer wavelengths such as visible or infrared light [28]. If not dissipated, the excess energy may lead to bond cleavage (photochemical degradation) [28]. This degradation contributes to deterioration of the molecule, causing dimensional changes, color and brightness changes, loss of opacity, crack formation, and hardening [28].

Despite of the increase in the dimensional changes in all groups, overall, the samples displayed excellent dimensional stability. The average of dimensional change in all groups remained within the standard recommended by ISO 4823 [32], which states that the contraction should not exceed 1.5% after 24 hours (tables 1 and 3).

With regard to detail reproduction, all groups scored 2 over the period analyzed. According to the present detail reproduction classification, level 2 means that all samples fully reproduced the 3 grooves with accurate angles [6]. These results are in agreement with Goiato et al. [6], who reported that silicones present excellent detail reproduction capacity, reproducing grooves of 20 μm.

The use of AutoCAD software as a method to evaluate the dimensional stability of facial silicones appears to be satisfactory, and produced accurate values. The applicability of this software in many areas of medicine and dentistry is unquestionable [23,24] and highlights the possible contributions through the association of computational methods with the health sciences.

The limitation of the present study relies on the mechanism of artificial aging used which differs from the natural aging mechanism to which medical silicones are normally subjected. During normal function, most elastomers used in facial prostheses are not exposed to the wet environments or thermal cycling procedures used during the artificial aging process. Although artificial weathering causes a greater change than natural weathering [13], there is no definitive research examining the correlation between the aging chamber used in the present study and clinical changes in physical properties of silicones. Future research is warranted to investigate this correlation.

## Conclusions

Incorporation of opacifiers alters the dimensional stability of facial prosthesis silicone, but seems to have no influence on detail reproduction. Accelerated aging is responsible for most of the dimensional changes in Silastic MDX4 4210, but all dimensional changes measured in this study remained within the limits of stability necessary for this application.

## Competing interests

The authors declare that this research was conducted without any commercial or financial relationships that could be construed as a potential conflict of interest.

## Authors' contributions

MCG participated in the design and coordination of the study and helped draft the manuscript. MFH conceived the study, fabricated the samples, participated in the sequence alignment, carried out portions of the chemical disinfection process, and drafted the manuscript. MACS performed the statistical analysis. DMS carried out the detail reproduction assays and the accelerated aging process. AAP carried out the dimensional stability assays. AM carried out part of chemical disinfection process and participated in interpretation of data. All authors read and approved the final manuscript.

## Authors' Information

MCG - Professor in charge of patient rehabilitation with maxillofacial prostheses at the Oral Oncology Center (COB) of the Aracatuba Dental School (FOA - UNESP) and coordinator of the research field "Aesthetics in Maxillofacial Prosthetic Materials".

MFH, DMS, AAP e AM - Trainees at the Oral Oncology Center (COB) of the Aracatuba Dental School (FOA - UNESP) and members of the research field "Aesthetics in Maxillofacial Prosthetic Materials"

MACS - Chair Professor of the Dental Materials division in the Department of Periodontology and Prosthesis at Piracicaba Dental School (FOP - UNICAMP).
